# A Clinical Case

**Published:** 1884-07

**Authors:** C. Lippe

**Affiliations:** New York


### CLINICAL CASE.

C. Lippe, M. D., New York.

H. B., æt. eleven, awoke in the morning feeling tired, pain in back and limbs. As the day progressed commenced to feel prostrated and the throat pained him. Was called to see the patient in the evening. Found him very much prostrated; pain in the back and limbs; legs from the knee down cold; dry, hot skin elsewhere; pulse, 120; throat on inspection showed an ulcerated spot on right tonsil, tongue in spots coated a yellowish white, breath offensive, putrid; on swallowing the pain is at one time on the right side and again on the left side. Gave Lac. can.$^{cm}$ (Fincke), one dose. Slept some during the night and had a profuse perspiration. On my visit in the evening found a new condition of the case. The odor from the mouth was much less; pulse, 100; the acute pain less but a condition of quinsy had set in. Complained of a lump in the throat which prevented swallowing. On the following morning the same conditions prevailed. In the evening found the aggravation was on the morning on waking, and at 5 P. M. there was decided motion of the alæ nasi on breathing and the acute pain was referred entirely to the right side of the throat, although the swelling of tonsils and hard and soft palate made swallowing difficult. Acids could be swallowed without difficulty. Gave Lyc.$^{cm}$ (Fincke), one dose. Had a restless night. Jumping up and talking in his sleep. Pulse going slowly down. Let the Lyc. act for forty-eight hours. At first there was an amelioration, then all curative action ceased; the throat was still painful on the right side and sensitive to touch, but now a complaint is made that all food tastes of *kerosene*, not water. I had met this peculiar symptom in the case of a young lady who took the remedy and complained to me of this strange taste. Having some other symptoms to warrant me, one dose of Lach.$^{cm}$ (Fincke) was given in the evening. The night was followed by a quiet sleep, and on the following morning found but little swelling, no ulceration. One more visit was paid and the case dismissed.

---

